# Cyclooxygenase-2 Facilitates Newcastle Disease Virus Proliferation and Is as a Target for Canthin-6-One Antiviral Activity

**DOI:** 10.3389/fmicb.2020.00987

**Published:** 2020-05-20

**Authors:** Chongyang Wang, Ting Wang, Ruochen Hu, Jiangkun Dai, Haijin Liu, Na Li, Uwe Schneider, Zengqi Yang, Junru Wang

**Affiliations:** ^1^College of Chemistry and Pharmacy, Northwest A&F University, Yangling, China; ^2^College of Veterinary Medicine, Northwest A&F University, Yangling, China; ^3^College of Food Science and Technology, Northwest University, Xi’an, China; ^4^School of Chemistry, The University of Edinburgh, Edinburgh, United Kingdom

**Keywords:** cyclooxygenase-2, prostaglandin E_2_, Newcastle disease virus, canthin-6-one, antiviral

## Abstract

Cyclooxygenase-2 (COX-2), one of the mediators of inflammation in response to viral infection, plays an important role in host antiviral defense system. But its role in Newcastle disease virus (NDV) proliferation process remains unclear. This study revealed that inhibition of COX-2 could benefit NDV proliferation and overexpression of COX-2 dose-dependently suppressed NDV proliferation. Overexpression of COX-2 also showed inhibitory effect on NDV-induced endoplasmic reticulum (ER)-stress and autophagy, also promoted the expression of antiviral genes. However, prostaglandin E_2_ (PGE_2_), the major product of COX-2, had indistinctive effects on NDV proliferation. At variant time point post viral infection, a tight regulation pattern of COX-2 by NDV was observed. Using inhibitors and siRNA against signaling molecules, the nuclear factor-κB (NF-κB) and melanoma differentiation-associated gene 5 (MDA5) were identified as critical factors for NDV induced COX-2 expression. Nonetheless, at late stage of NDV proliferation, substantial suppression of COX-2 protein synthesis could be detected, accompanied by a decrease in mRNA half-life. Furthermore, three C ring-truncated canthin-6-one analogs were used to activate COX-2 expression and showed inhibitory effect on NDV proliferation with the effective concentrations on μM level. Taken together, these results illustrated a novel NDV-regulated cellular mechanism and indicated that COX-2 is an important regulator of NDV proliferation which can serve as a potential target for anti-NDV agents.

## Introduction

Newcastle disease (ND) is caused by the ND virus (NDV), which is a highly contagious pathogen among avian species causing huge economic losses in commercial and backyard poultry (Alexander, 2000). NDV belongs to the genus *Avulavirus* of the family *Paramyxoviridae* and contains a single-stranded negative-sense RNA genome, which encodes six structural proteins, including: nucleocapsid protein (NP), phosphoprotein (P), matrix protein (M), fusion protein (F), hemagglutinin-neuraminidase protein (HN), and the large polymerase protein (L) (Cox and Plemper, 2017).

During infection with NDV, viral RNA (vRNA) is sensed by pattern-recognition receptors (PRRs) such as the melanoma differentiation-associated gene 5 (MDA5), which belongs to the RIG-I-like receptor (RLR) family (Motz et al., 2013). During NDV infection, various signaling pathways are stimulated, it was also shown that the NDV virus was able to stimulate rapid and strong innate immune and pro-inflammatory responses (Kang et al., 2015). Among these, the cyclooxygenase (COX) enzyme plays an important role as part of the pro-inflammatory response (Gilroy et al., 1999).

The COX enzyme, also known as prostaglandin (PG) H/G synthase, is the rate-limiting enzyme that converts arachidonic acid into PGs (Rumzhum and Ammit, 2016). COX-1 is considered as a “housekeeping” enzyme. Meanwhile, the major functional isoform, COX-2, is reported to be associated with inflammation, cancer, autophagy, and viral infection (Gilroy et al., 1999; Zelenay et al., 2015; Dudek et al., 2016; Niranjan et al., 2018). During influenza A virus (IAV) infection, COX-2 expression was shown to be tightly regulated and to exhibit anti-IAV activity (Dudek et al., 2016). However, COX-2 gene silencing and catalytic inhibition were shown to sufficiently suppress dengue virus (DENV) proliferation (Lin et al., 2017), which indicated the function of COX-2 to be diverse during infection of different viruses.

One of the important products of COX-2-triggered catalysis, prostaglandin E_2_ (PGE_2_), is a bioactive lipid with a broad range of biological effects associated with inflammation, cancer, and antiviral immunity (Coulombe et al., 2014). PGE_2_ was identified as an inhibitor of type I interferon (IFN) in macrophages. Similarly, the addition of exogenous PGE_2_ displayed opposing effects on different virus infections. During IAV infection, the addition of PGE_2_ decreased IAV proliferation (Dudek et al., 2016), whereas during DENV infection, the viral titers of PGE_2_-treated cells were increased (Lin et al., 2017).

Canthin-6-one alkaloids, a subclass of β-carboline, were first isolated in 1952 from the Australian tree *Pentaceras australis* (Nelson and Price, 1952). These kinds of alkaloids were shown to have broad biological activity, such as antitumor, anti-inflammatory, antibacterial, and antiviral (Dai et al., 2016). However, the antiviral mechanism of these compounds was rarely studied. Recent years, our group has synthesized more than 50 canthin-6-one analogs, some of them had the ability to inhibit bacteria (Dai et al., 2018a, b). Among these analogs, C-ring truncated alkaloids showed the best antibacterial activity through damaging bacterial cell membranes and influencing the membrane formation (Dai et al., 2018b). Our previous study also showed these analogs could affect the expression of COX-2 in RAW264.7 cells (unpublished).

To date, however, the role of COX-2 or PGE_2_ in NDV proliferation has remained unclear. In order to confirm the correlation between COX-2 and NDV, we investigated the effect of COX-2 and PGE_2_ on NDV proliferation, respectively. In this context, we examined the regulation of COX-2 upon NDV infection and the mechanism of COX-2 alteration. Three C ring-truncated canthin-6-one analogs were identified as anti-NDV compounds via induced COX-2 expression.

## Materials and Methods

### Cell Lines, Viruses

DF-1 cells and BHK-21 cells originally obtained from ATCC (Manassas, VA, United States) were purchased from Cell Bank of Chinese Academy Sciences (Shanghai, China). DF-1 cells and BHK-21 cells were cultured in Dulbecco’s modified Eagle’s medium (DMEM; Gibco, United States) supplemented with 10% fetal bovine serum (FBS; Gibco, United States) at 37°C with 5% CO_2_.

Two NDV strains, including F48E9, PPMV-1/SX-01/Ch/15 (SX01), were provided by College of Veterinary Medicine, Northwest A&F University (Yangling, China).

### Chemicals and Antibodies

NS-398, celecoxib, MG-132, PGE_2_, and actinomycin D were purchased from MedChemExpress (MCE, United States). C ring-truncated canthin-6-one analogs were synthesized as previously described (Dai et al., 2018b). All compounds were dissolved in dimethyl sulfoxide (DMSO) for *in vitro* studies.

### Plaque Assay

DF-1 cells were seeded in 24-well plates for 24 h. Then cells were infected with samples with dilution of 10^–1^–10^–5^. After adsorption at 37°C for 1 h, unbound virions were removed by washing with phosphate buffer saline (PBS). DF-1 cells were covered with medium containing methyl cellulose (1%). The cells were fixed and stained with crystal violet solution for 30 min. Plaques were visualized and virus titer was calculated.

### Cytotoxicity Assay

The cytotoxicity of all compounds was measured using a commercial CCK-8 kit (Beyotime, China) according to the manufacturer’s instructions. Briefly, DF-1 cells were seeded in 96-well plates and cultured for 24 h before serial concentrations of compounds or inhibitors were added in triplicate. After 48 h, 10 μl CCK-8 reagent was dispensed into each well. And the plates were incubated for additional 2 h. The optical density was measured at 450 nm (Model 680 Microplate Reader, Bio-Rad, United States).

### Plasmid Construction, siRNA, and Transient Transfection

The full-length COX-2 expression plasmid was created by PCR amplification of COX-2 cDNA (NCBI number: NM_001167719). Primers and siRNA used in this study are listed in Table 1. DF-1 cells were transfected with TurboFect transfection reagent (Thermo Scientific, United States) according to the manufacturer’s instructions.

**TABLE 1:** List of primers and siRNA used in this study.

Primer or siRNA	Sequence (5′–3′)	Purpose
COX-2-F	ATGCTGCTGCCTTGTGCACTGCTGG	Gene clone
COX-2-R	TTACAACTCAGCAGATTGCTCTTTC	Gene clone
qCOX-2-F	AATATGCTCCCCTGAGTAACTGG	qPCR
qCOX-2-R	ACAGCCTTCACATTGTTGC	qPCR
qβ-actin-F	GTCTTCACCACCATGGAGAA	qPCR
qβ-actin-R	ATGGCATGGACTGTGGTCAT	qPCR
qF48E9-F	TCATGCCCAGCTACCTGTCG	qPCR
qF48E9-R	CTGTTGGATTTCAGACCGCATC	qPCR
qSX01-F	CAGCCACCCGCATCCGAGCAG	qPCR
qSX01-R	GGTTGTTTCCACGGCTCGACT	qPCR
qIFN-α-F	GACATGGCTCCCACACTACC	qPCR
qIFN-α-R	AGGCGCTGTAATCGTTGTCT	qPCR
qIFN-β-F	GCTCACCTCAGCATCAACAA	qPCR
qIFN-β-R	GGGTGTTGAGACGTTTGGAT	qPCR
qMX1-F	AAGCCTGAGCATGAGCAGAA	qPCR
qMX1-R	TCTCAGGCTGTCAACAAGATCAA	qPCR
qOASL-F	AGATGTTGAAGCCGAAGTACCC	qPCR
qOASL-R	CTGAAGTCCTCCCTGCCTGT	qPCR
siNC	UUCUCCGAACGUGUCACGUTT	RNA interference
siMDA5	GGUAUCAAGUUAUUGGCUUTT	RNA interference

### RNA Extraction and Quantitative Real-Time PCR

Total cellular RNA was extracted using TRIzol (Invitrogen, United States) according to the manufacturer’s instructions. Quantitative real-time PCR (qPCR) was conducted using 2 × RealStar green power mixture (GenStar, China) according to the manufacturer’s instruction. Relative expressions of target gene levels were calculated by using 2^–Δ^ ^Δ^ ^CT^ as previously described (Livak and Schmittgen, 2001). Samples were normalized to the quantity of β-actin gene. The primers used in this study are listed in Table 1.

### Western Blot Analysis

Cells were washed twice with PBS and lyzed with RIPA lysis buffer (Solarbio, China) supplemented with protease inhibitors on ice for 30 min. Denatured cell lysates were subjected to sodium dodecyl sulfate-polyacrylamide gel electrophoresis (SDS-PAGE) and then transferred to polyvinylidene difluoride (PVDF) membranes (Millipore, United States). PVDF membranes were immunoblotted with primary antibodies against β-actin, COX-2, NP, or HA flag followed by HRP conjugated secondary antibody. The immunostained proteins were visualized using an ECL peroxidase substrate (Millipore, United States) detection reagent system. The primary antibodies used in this study included antibodies against β-actin, COX-2, and HA-flag (Cell Signaling Technology, United States). The mAb against NDV NP was provided by College of Veterinary Medicine, Northwest A&F University (Yangling, China).

### PGE_2_ ELISA

DF-1 cells were incubated with COX-2 inhibitors at the indicated concentrations. At 24 h post incubation, the supernatant was harvested, and measured with the ELISA kit (Cayman Chemical, Ann Arbor, MI, United States) according to the manufacturer’s instructions.

### Statistical Analysis

The data were represented as means ± standard deviation (SD) of at least three independent experiments. Significance was analyzed with two-tailed Student’s *t*-test using GraphPad Prism 6.0 software (United States). Statistical significance: ns, not significant, ^∗^*p* < 0.05, ^∗∗^*p* < 0.01, and ^∗∗∗^*p* < 0.001.

## Results

### NDV Proliferation Is Affected by COX-2 and Its Inhibitors

**FIGURE 1:**
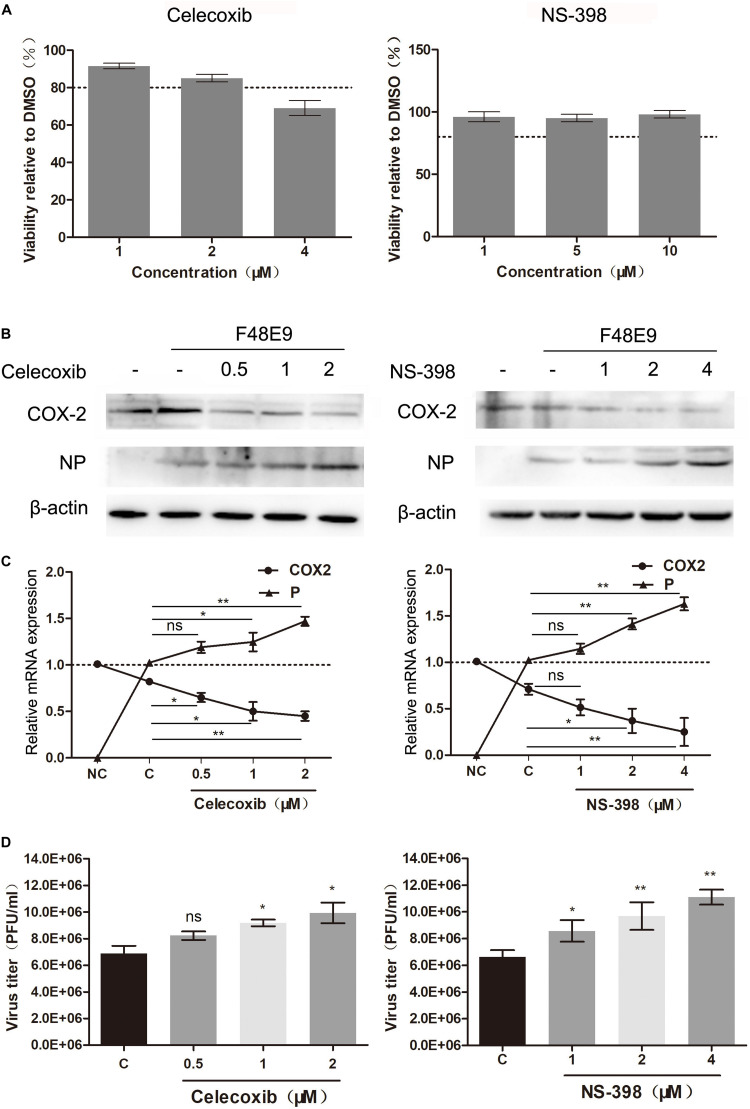
Enhanced proliferation of the Newcastle disease virus through inhibition of COX-2 by inhibitors. **(A)** DF-1 cell viability was determined using CCK-8 assay after incubated with the COX-2 inhibitors Celecoxib (left) and NS-398(right) for 48 h, respectively. **(B–D)** DF-1 cells were infected with the F48E9 (MOI = 0.01) and incubated in the absence or presence of the corresponding inhibitor for 24 h. Cell lysates were subjected to western blot **(B)**. Relative mRNA expression was analyzed by qPCR **(C)**. The virus titer in the supernatant was determined by the plaque assay **(D)**. The data are representative of the mean ± SD of three independent experiments. Statistical significance was assessed using two-tailed Student’s *t*-test. ns, not significant; *p < 0.05; **p < 0.01.

In order to examine the effect of COX-2 inhibitors on NDV proliferation, two COX-2 inhibitors, NS-398 and Celecoxib, were used based on the cell viability assay data (Figure 1A). NS-398 and celecoxib both can suppress the expression level of PGE_2_, which suggested that COX-2 activity was inhibited (Supplementary Figure S2A).

DF-1 cells were infected with F48E9 and incubated with NS-398 and Celecoxib (various concentrations). At 24 h post infection, incubation of celecoxib (2 μM) or NS-398 (4 μM) resulted in considerably increased viral titers compared with the control group (1.54-fold and 1.67-fold, respectively) (Figure 1D). The total cell lysates and cellular RNA were extracted and subjected to western blot and qPCR, respectively. Incubation of celecoxib or NS-398 also leads to higher expression level of viral protein and mRNA (Figures 1B,C). Similar result was observed in BHK-21 cells. NS-398 also showed enhancing effect on NDV proliferation in BHK-21 cell line (Supplementary Figure S1). Taken together, these results indicated that COX-2 inhibitors dose-dependently enhanced NDV proliferation.

**FIGURE 2:**
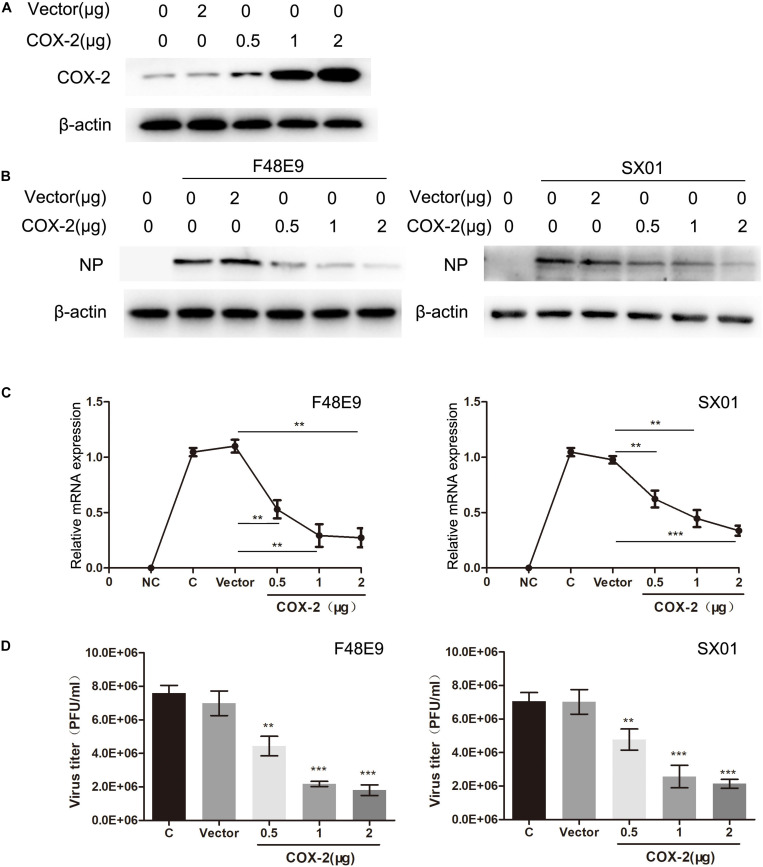
Decreased NDV proliferation through overexpression of COX-2. **(A)** DF-1 cell lysates were subjected to western blot after transfected with pCAGGS or pCAGGS-COX-2 for 24 h. **(B–D)** DF-1 cells were transfected with pCAGGS or pCAGGS-COX-2 for 24 h prior to infection with F48E9 (left) or SX01 (right) at an MOI of 0.01. At 24 h post infection, cell lysates were subjected to western blot **(B)**. Relative mRNA expression was analyzed by qPCR **(C)**. The virus titer in the supernatant was determined by the plaque assay **(D)**. The data are representative of the mean ± SD of three independent experiments. Statistical significance was assessed using two-tailed Student’s *t*-test. **p < 0.01; ***p < 0.001.

In order to assess the effect of exogenous COX-2 on NDV proliferation, we constructed eukaryotic expression vector which can express COX-2. Figure 2 shows that overexpression of COX-2 decreased the viral titers in a concentration-dependent manner. When DF-1 cells were transfected with COX-2 at the concentration of 2 μg, viral titers of F48E9 or PPMV were decreased to 17 or 26% of the control group, respectively. The inhibitory effect could also be verified through the levels of viral protein and vRNA (Figure 2). And two strains of NDV were used in our study, which means that the antiviral effect was not strain-specific. Taken together, the inhibition of COX-2 could enhance the proliferation of NDV, and the exogenous COX-2 expression could inhibit NDV proliferation.

As a main product of COX-2, we further studied the effect of PGE_2_ on NDV proliferation. DF-1 cells infected with NDV were then incubated with PGE_2_. The results revealed that NDV proliferation is not affected by the addition of exogenous PGE_2_ (Supplementary Figure S3).

### COX-2 Inhibits NDV-Induced Autophagy and Promotes the Type I IFN Responses

**FIGURE 3:**
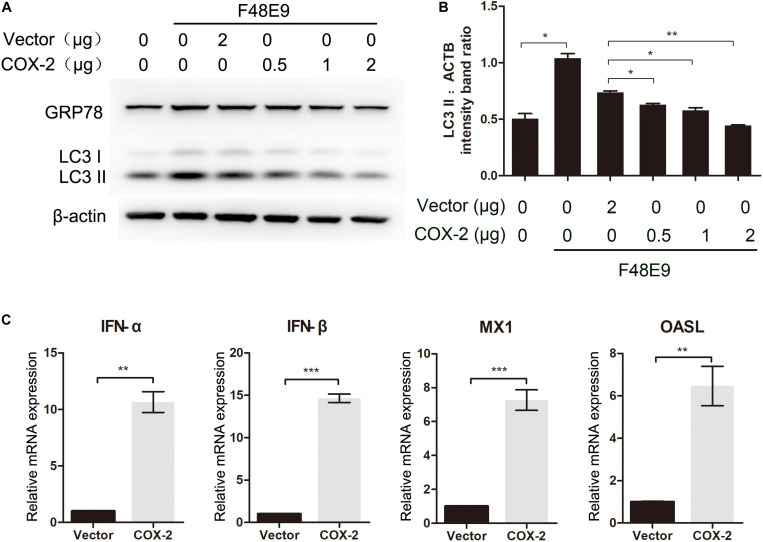
Influence of COX-2 expression on NDV-induced autophagy and the expression of antiviral genes. **(A,B)** DF-1 cells were transfected with pCAGGS or pCAGGS-COX-2 for 24 h prior to infection with F48E9 at an MOI of 0.01. At 24 h post infection, cell lysates were subjected to western blot **(A)**. The LC3-II levels relative to the ACTB levels were determined by densitometry **(B)**. DF-1 cells were transfected with pCAGGS (2 μg) or pCAGGS-COX-2 (2 μg) for 24 h prior to infection with F48E9 at an MOI of 0.01. At 24 h post infection, relative mRNA expression was analyzed by qPCR **(C)**. The data are representative of the mean ± SD of three independent experiments. Statistical significance was assessed using two-tailed Student’s *t*-test. ^∗∗^*p* < 0.01; ^∗∗∗^*p* < 0.001.

Taken all data into account, these data suggested that antiviral effect of COX-2 was not the result of PGE_2_ induction. Sun et al. (2014) reported that NDV-induced autophagy could promote its proliferation in cells and tissues. To characterize mechanisms of effect of COX-2 during NDV infection, we determined if COX-2 inhibited NDV-induced autophagy. DF-1 cells were transfected with COX-2 or vector. At 24 h post transfection, DF-1 cells were then infected with F48E9 for another 24 h. The results showed that exogenous COX-2 significantly reduced the increased formation of LC3-II induced by NDV infection (Figures 3A,B). Meanwhile, glucose-regulated protein 78 (GRP78), as a marker protein of endoplasmic reticulum (ER)-stress (Cheng et al., 2016), was also decreased, which indicated that COX-2 may exert the antiviral effect through inhibition of NDV-induced ER-stress and autophagy.

Autophagy is reported to be associated with the regulation of the expression of type I IFN (Tian et al., 2019). Therefore, to gain the better insights into the COX-2-mediated inhibition of NDV, we examined the expression level of type I IFN and IFN-stimulated genes (ISGs). As shown in Figure 3C, exogenous COX-2 expression significantly enhanced the expression of type I IFN and ISGs. Treatment with NS-398 reduced the expression of these genes (Supplementary Figures S2B–E). These findings suggest that COX-2 expression shows inhibitory effect on NDV-induced autophagy and enhancing effect on IFN expression.

### Induction of COX-2 Expression at the Middle Stage of NDV Infection Is Mediated by NF-κB and MDA5

**FIGURE 4:**
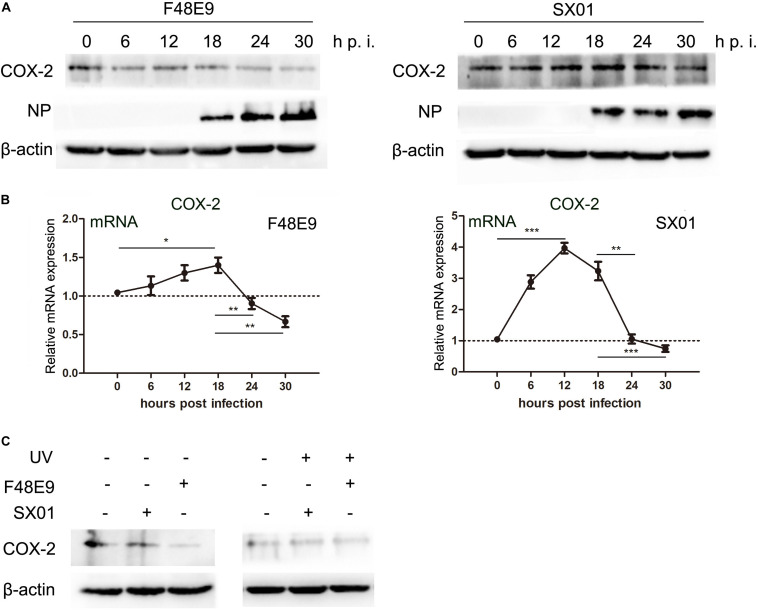
Influence of NDV infection on COX-2 expression. **(A,B)** DF-1 cells were infected with 0.01MOI of F48E9 (left) or SX01 (right) for the indicated times post infection, respectively. Cell lysates were subjected to western blot **(A)**. Relative mRNA expression was analyzed by qPCR **(B)**. **(C)** DF-1 cells were infected with UV-inactivated NDV or replicating virus at an MOI of 0.01. At 30 h post infection, cell lysates were subjected to western blot. The data are representative of the mean ± SD of three independent experiments. Statistical significance was assessed using two-tailed Student’s *t*-test. *p < 0.05; **p < 0.01; ***p < 0.001.

In order to further investigate the regulation of COX-2 during NDV infection, we measured the mRNA and protein levels of COX-2 in NDV-infected DF-1 cells by western blot and qPCR, respectively. At 12 and 18 h post infection, the highest mRNA levels of COX-2 were observed (Figure 4B). Meanwhile, the protein levels were also higher than 6 h post infection (Figure 4A). These results indicated that NDV induced COX-2 expression in a well time-controlled manner.

**FIGURE 5:**
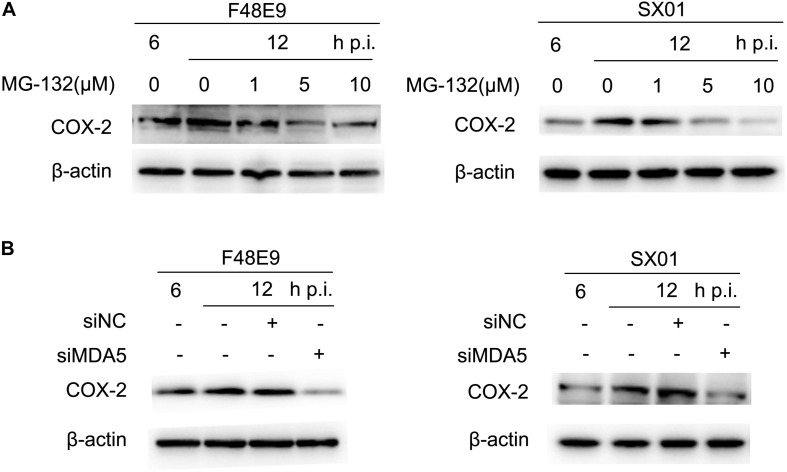
NDV-mediated induction of COX-2 via NF-κB and MDA5. **(A)** DF-1 cells were infected with 0.01MOI of F48E9 (left) or SX01 (right) for 6 h prior to incubation with MG-132, and cell lysates were harvested at indicated times post infection. **(B)** DF-1 cells were transfected with siNC or siMDA5 for 24 h prior to infection with 0.01 MOI of F48E9 (left) or SX01 (right). At different time points post infection, cell lysates were prepared and subjected to western blot.

RIG-I is reported to involve in regulation of COX-2 expression (Dudek et al., 2016). Due to the lack of RIG-I in chickens, we investigated whether the closest relative of RIG-I, MDA5, could play a role in COX-2 expression (Mitsutoshi et al., 2005; Cheng et al., 2015). siMDA5 could significantly inhibit the exogenous expression of MDA5 (Supplementary Figure S5). siNC was used as the negative control which is silence for non-sense sequence. Transfection of siMDA5 triggered a significant decrease of NDV-induced COX-2 expression at the protein level (Figure 5B), which suggested that MDA5 is essential for NDV-induction of COX-2.

NF-κB was identified as an important regulator of COX-2 in response to dsRNA or viral infection in macrophages (Steer et al., 2003). In order to identify the regulatory mechanism of NDV-induced COX-2 expression, the effect of MG-132 (an NF-κB inhibitor) on NDV-induced COX-2 expression was analyzed by western blot. MG-132 was shown to dose-dependently decrease the protein levels of COX-2 induced by NDV infection (Figure 5A). Thus, these data suggest that NF-κB is apt to operate as mediators for NDV-induced COX-2 expression.

### Decreased COX-2 Expression During Later Stages of NDV Infection Is Accompanied With Loss of mRNA Stability

**FIGURE 6:**
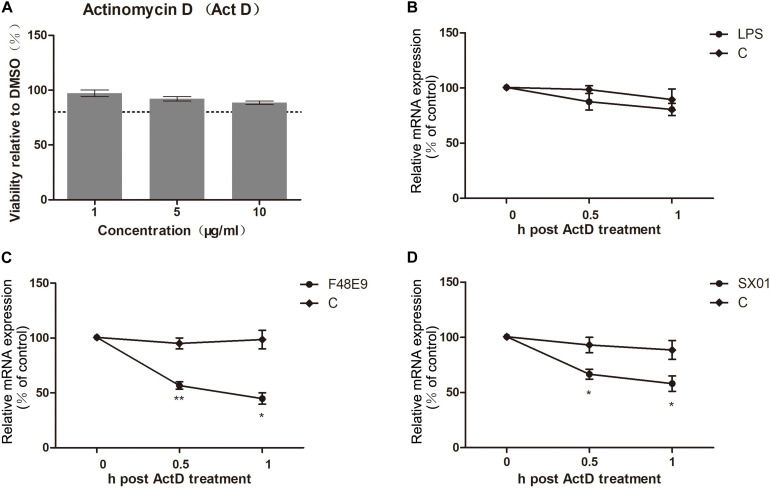
Loss of COX-2 mRNA stability. **(A)** DF-1 cell viability was determined after incubated with actinomycin D (Act D) for 2 h at the indicated concentrations. **(B)** DF-1 cells were incubated with 1 μg LPS for 4 h prior to incubation with Act D (3 μg/ml). At different time points post incubation, cellular RNA was analyzed by qPCR. **(C,D)** DF-1 cells were infected with 5 MOI of F48E9 **(C)** or SX01 **(D)** prior to incubation with Act D (3 μg/ml). At different time points post incubation, relative mRNA expression was analyzed by qPCR. The data are representative of the mean ± SD of three independent experiments. Statistical significance was assessed using two-tailed Student’s *t*-test. *p < 0.05; **p < 0.01.

While the COX-2 expression was induced by NDV at about 12–18 h post infection (compared to 6 h post infection), expression of COX-2 was significantly decreased at later stages (24–30 h post infection). Suppression of COX-2 expression during NDV proliferation was also observed in BHK-21 cells (Supplementary Figures S4A,B). Moreover, the reduction of COX-2 was only found in cells infected with live virus, stimulation with UV-inactivated NDV was unable to decrease the expression of COX-2 (Figure 4C). Since the NDV infection resulted in a decrease of COX-2 mRNA quantities in DF-1 cells, we measured the COX-2 mRNA half-life. Actinomycin D (Act D), which inhibits both the transcription/initiation by intercalating into DNA and the formation of novel mRNA, was used in this study (Hidenori and Nobuyoshi, 2012). Viability of Act D was tested by CCK-8 assay (Figure 6A). DF-1 cells infected with NDV were subsequently treated with Act D; RNA was extracted at indicated time points and analyzed by qPCR. NDV infection caused a significant decline of COX-2 mRNA which indicated a reduced mRNA stability (Figures 6C,D). Meanwhile, lipopolysaccharide (LPS) was used in control experiment. LPS treatment did not cause significant differences in COX-2 mRNA stability (Figure 6B). Taken together, these results indicate the NDV-mediated effect on COX-2 mRNA stability.

### Inducer of COX-2 Decreased NDV Proliferation

**FIGURE 7:**
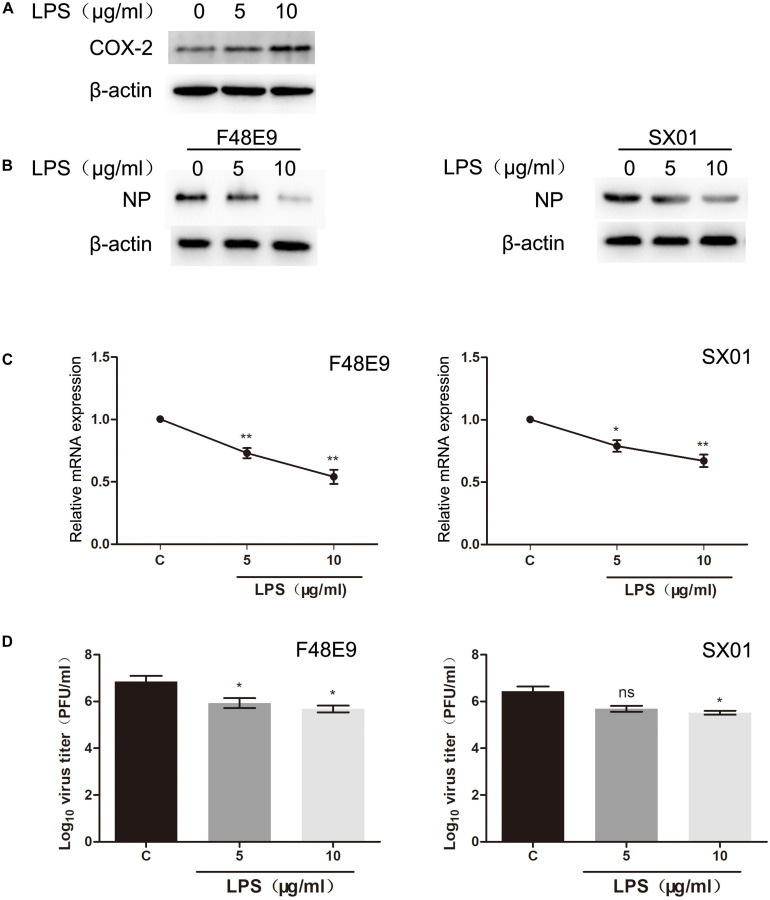
Decreased proliferation of NDV through LPS treatment. **(A)** DF-1 cells were incubated with LPS at indicated concentrations. At 24 h post incubation, cell lysates were subjected to western blot. **(B–D)** DF-1 cells were incubated with LPS for 24 h prior to infection with 0.01 MOI of F48E9 (left) or SX01 (right). At 24 h post infection, cell lysates were subjected to western blot **(B)**. Relative mRNA expression was analyzed by qPCR **(C)**. The virus titer in the supernatant was determined by the plaque assay **(D)**. The data are representative of the mean ± SD of three independent experiments. Statistical significance was assessed using two-tailed Student’s *t*-test. ns, not significant; *p < 0.05; **p < 0.01.

Lipopolysaccharide is a well-known inducer of COX-2 (Kirkby et al., 2013). To evaluate whether LPS facilitates NDV proliferation, we pre-treated DF-1 cells with different concentrations of LPS. After 24 h post treatment, COX-2 were significantly stimulated by LPS (10 μg/ml) (Figure 7A). Then, cells were infected with NDV (F48E9 or SX01). At 24 h post infection, viral titer, mRNA, and NP protein were determined. As shown in Figure 7, LPS (10 μg/ml) significantly increased the expression of COX-2 and decreased NDV proliferation.

**FIGURE 8:**
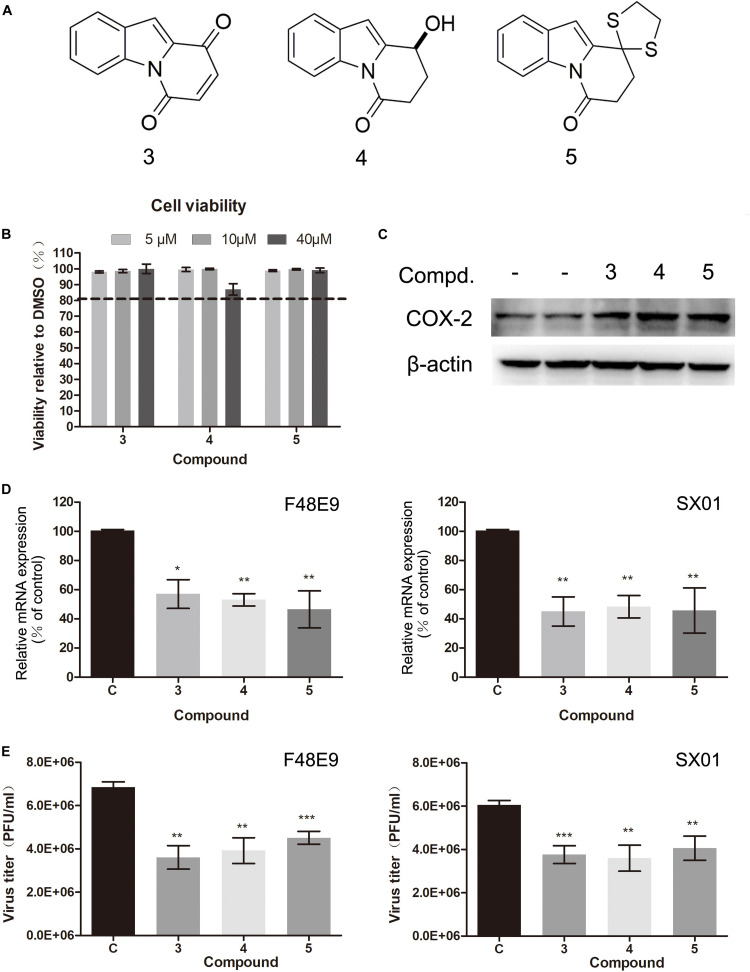
Effect of C ring truncated canthin-6-one analogs on COX-2 expression and NDV proliferation. **(A)** The molecular structure of the three C ring-truncated canthin-6-one analogs. **(B)** DF-1 cell viability was determined using CCK-8 assay after incubated with the compounds for 48 h. **(C)** DF-1 cells were incubated with the compounds (10 μM). At 24 h post incubation, cell lysates were subjected to western blot. **(D,E)** DF-1 cells were infected with 0.01 MOI of F48E9 (left) or SX01 (right) and incubated in the absence or presence of the corresponding compounds for 24 h. Relative mRNA expression was analyzed by qPCR **(D)**. The virus titer in the supernatant was determined by the plaque assay **(E)**. The data are representative of the mean ± SD of three independent experiments. Statistical significance was assessed using two-tailed Student’s *t*-test. *p < 0.05; **p < 0.01; ***p < 0.001.

In our previous study, C ring-truncated canthin-6-one analogs were identified as activators of COX-2 in RAW264.7 cells (unpublished). Their structures are shown in Supplementary Table S1. In this study, their effect on COX-2 expression was studied. Their molecular structures are presented in Figure 8A. We investigated whether these compounds could inhibit NDV proliferation. Viability of these compounds was tested by CCK-8 assay. They all showed low toxicity and increased COX-2 expression (Figures 8B,C). Furthermore, these compounds significantly reduced NDV proliferation (Figures 8D,E). Among them, compound 3 is the best inhibitor of NDV, the amount of virus could reduce 40–52% compared to the control group (Figure 8E). These results indicate that inducer of COX-2 could inhibit NDV proliferation. And C ring-truncated canthin-6-one analogs may serve as potential anti-NDV agents.

## Discussion

COX-2 and its major product (PGE_2_) were reported to have diverse effects on virus proliferation. COX/PGE_2_ signaling pathway showed enhancing effects on human cytomegalovirus, dengue virus, vesicular stomatitis virus, pseudorabies virus, and so on (Chen et al., 2002; Neelanjana et al., 2004; Schroer and Shenk, 2008; Lin et al., 2017). Meanwhile, on human immunodeficiencyvirus-1, adenovirus, measles virus, and parainfluenza 3 virus, COX/PGE_2_ signaling pathway exhibited antiviral effects (Luczak et al., 1975; Dore-Duffy, 1982; Ongrádi et al., 1994; Hayes et al., 2002). In the present study, we observed that inhibition of COX-2 expression was beneficial to NDV proliferation in DF-1 cells and BHK-21 cells.

During virus infection, the first defense line of non-specific immune response is generated by sensing NDV vRNA via PRRs, such as RIG-I-like Receptor (RLR) family (Osamu and Shizuo, 2010). Mibayashi et al. reported RIG-I plays a role in expression of COX-2 in endothelial cells (Masaki et al., 2007). To our knowledge, MDA5, which share a number of structural similarities with RIG-I, its function in expression of COX-2 was not investigated to date. Indeed, our data confirmed that MDA5 as the mediator for the NDV-induced COX-2 expression. Due to the vRNA sensing function of MDA5, we also supposed that NDV RNA could directly stimulate the COX-2 expression.

After virus is recognized by MDA5, downstream signaling is initiated via the interaction between MDA5 and mitochondrial antiviral-signaling protein (MAVS). Subsequently, IκB kinases (IKKs) and TANK-binding kinase 1 (TBK1) form a complex with MAVS, leading to the activation of IFN regulatory factor 3 (IRF3) and nuclear factor-κB (NF-κB) pathways and drives expressions of extensive downstream genes (Hou et al., 2011). COX-2 expression was also reported to be affected by the inhibition of NF-κB in RAW 264.7 macrophages (Steer et al., 2003). It is consistent with the results obtained from our data. NF-κB inhibitors could prevent the virus-induced COX-2 expression which indicated that COX-2 upregulation is relied on the activation of NF-κB pathways.

After a short period of upregulation, COX-2 expression is eventually decreased. As COX-2 synthesis is a complex process regulated at many levels including transcriptional, post-transcriptional, or post-translational (Rumzhum and Ammit, 2016). From the data shown in Figure 4, COX-2 mRNA and protein levels were consistent. While mRNA level was reduced at 24 h post infection, protein level showed similar trends, which indicated the protein level is associated with mRNA of COX-2. And this association suggested that COX-2 downregulation is directly due to the decreasing mRNA of COX-2. Since COX-2 was reported to increase angiogenesis through induction of vascular endothelial growth factor (VEGF), and COX-2 inhibitors could limit tumor growth (Masferrer et al., 2000; Riedl et al., 2004). Meanwhile, NDV is one kind of the oncolytic viruses with great potential (Meng et al., 2012). Therefore, we suggested that NDV may exhibit the oncolytic effect through downregulating of COX-2.

Downregulation of COX-2 was reported to initiates autophagy and promotes 1-methyl-4-phenyl 1,2,3,6 tetrahydropyridine (MPTP)-induced cell death (Niranjan et al., 2018). Recent years, many studies focused on the effect of autophagy on viral infection (Sun et al., 2012; Hu et al., 2015). We considered to investigate whether autophagy involved in the COX-2-inhibition of NDV. NDV was previously reported to induce autophagy through ER stress-related unfolded protein response (UPR) pathways to benefit its proliferation, and inhibition of autophagy could decrease NDV proliferation (Sun et al., 2014; Cheng et al., 2016). In the present study, we found that exogenous COX-2 could inhibit the NDV-induced ER-stress and autophagy. Nevertheless, the mechanism of how the autophagy functions as regulator of NDV proliferation still remained unknown. Recent years, with the deepening of research, autophagy is reported to play an important role in antiviral immune responses and involved in the regulation of the expression of type I IFN (Tian et al., 2019). Human parainfluenza virus type 3, another member of the *Paramyxoviridae* family, could induce mitophagy, subsequently inhibiting the type I IFN response via degrading MAVS (Ding et al., 2017). Here, we found that exogenous expression of COX-2 simultaneously promoted the expression of type I IFN and ISGs. Thus, the type I IFN and ISGs induced by COX-2 expression might have been due to the inhibition of autophagy. However, the connection between IFN responses and autophagy during the NDV infection process needs further study.

As a most important product of COX-2, PGE_2_ was well documented during several viruses proliferation process. Interestingly, controversial results were reported in influenza virus proliferation. Inhibition of PGE_2_ was reported to enhance antiviral responses by promoting the expression of type I IFN in macrophages (Coulombe et al., 2014). The exogenous PGE_2_ could inhibit IFN production mediated by the PGE_2_ receptors EP2 and EP4 (Full and Gack, 2014). Meanwhile, PGE_2_ treatment resulted in decreased viral titers in A549 cells (Dudek et al., 2016). However, when exogenous PGE_2_ was added during NDV infection, its proliferation was not affected compared to control group. Considering that COX-2 has more products, such as prostacyclin (PGI), prostaglandin D_2_ (PGD_2_), prostaglandin F_2__α_ (PGF_2α_), and thromboxane (Ricciotti and FitzGerald, 2011; Rumzhum and Ammit, 2016). Maybe these prostanoids could exert antiviral activity, or COX-2 could directly exhibit antiviral activity through other pathways.

As exogenous expression of COX-2 could inhibit NDV proliferation, other inducer of COX-2 may also have effect on NDV. In this study, LPS and several C ring-truncated canthin-6-one analogs were identified as inducer of COX-2 and inhibitor of NDV proliferation. However, also stimulates many other genes and lead to inflammation (Zhang et al., 2010). It was reported LPS treatment resulted in the induction of host’s adaptive immune response to inhibit influenza virus proliferation (Short et al., 2014). LPS treatment also stimulates RIG-I in endothelial cells (Tadaatsu et al., 2002). Thus, we could not ensure whether its activity was just the result of COX-2 induction. Same as LPS, canthin-6-one alkaloids showed multiple biological activity, such as inhibiting Wnt signaling and interfering with the G_2_/M transition (Camille et al., 2014; Kensuke et al., 2015). The cause of its antiviral activity may be not just because of COX-2 induction. All we can say is that COX-2 plays a role in their antiviral activity. The comprehensive mechanism of these compounds was still remained to be studied further. Meanwhile, according to the inhibition effect of COX-2 on influenza virus (Dudek et al., 2016), we speculated these C ring-truncated canthin-6-one analogs could serve as inhibitors of influenza virus, too. However, previous studies suggested that canthin-6-one alkaloids could exert anti-inflammatory effects and suppress the expression of COX-2, and these effects are the result of NF-κB inhibition (Tran et al., 2014; Cho et al., 2017). Paradoxically, in the present study, we showed these C-ring truncated analogs have opposite effect on inflammation. These results indicated that C-ring could be essential for its anti-inflammatory effect. C-ring may influence its inhibition effect on NF-κB.

## Conclusion

The effects of COX-2 on NDV proliferation were unraveled in DF-1 cells. Our data illustrated a tightly-regulated-pattern of NDV-regulated COX-2 expression and clarified the mechanism of COX-2 regulation by NDV. Moreover, three canthin-6-one analogs were identified as COX-2 inducer and inhibit NDV proliferation. These results provide a potential strategy for developing antiviral agents against NDV.

## Data Availability Statement

All datasets generated for this study are included in the article/Supplementary Material.

## Author Contributions

CW, TW, and RH carried out the experiments. CW collected data and wrote this manuscript. NL and JD provided chemical compounds and reagents. US and HL checked and revised the manuscript. NL, ZY, and JW conceived the study and participated in its design and coordination.

## Conflict of Interest

The authors declare that the research was conducted in the absence of any commercial or financial relationships that could be construed as a potential conflict of interest.
