# Supplementary material for: Cyclooxygenase-2 Facilitates Newcastle Disease Virus Proliferation and Is as a Target for Canthin-6-One Antiviral Activity
Source: Front Microbiol. 2020 May 20;11:987. doi: 10.3389/fmicb.2020.00987 (PMC7251056; doi:10.3389/fmicb.2020.00987)
Supplement: Supplementary file 1 [file Data_Sheet_1.docx]

Supplementary material

**Cyclooxygenase-2 Facilitates Newcastle Disease Virus**

**Proliferation and is as a Potential Target for Canthin-6-one Antiviral Activity**

*Chongyang Wang^1^, Ting Wang^2^, Ruochen Hu^2^ , Jiangkun Dai^1^,* *Haijin Liu^2^，Na Li^3^*，Uwe Schneider^4^ , Zengqi Yang^2^*, Junru Wang^1^**

*^1^College of Chemistry and Pharmacy, Northwest A&F University, Yangling, China，^2^College of Veterinary Medicine, Northwest A&F University, Yangling, China,* *^3^College of Food Science and Technology, Northwest University, Xi’an, China, ^4^School of Chemistry, The University of Edinburgh, Edinburgh, United Kingdom*

**Table S1** provided the molecular structure of six C ring-truncated canthin-6-one analogues.

**Figure S1** provided data for enhanced proliferation of NDV through inhibition of COX-2 in BHK-21 cell line.

**Figure S2** provided data for decreased expression of PGE_2_ and antiviral genes caused by NS-398 treatment.

**Figure S3** displayed the influence of PGE_2_ on NDV proliferation.

**Figure S4** showed the influence of NDV on COX-2 expression in BHK-21 cell line.

**Figure S5** showed the siMDA5 inhibit MDA5 expression.

**Table S1. The molecular structure of C ring-truncated canthin-6-one analogues.**

| Compound | Structure formula | MW | Compound | Structure formula | MW |
| --- | --- | --- | --- | --- | --- |
| 1 |  | 185 | 4 |  | 201 |
| 2 |  | 199 | 5 |  | 275 |
| 3 |  | 197 | 6 |  | 187 |


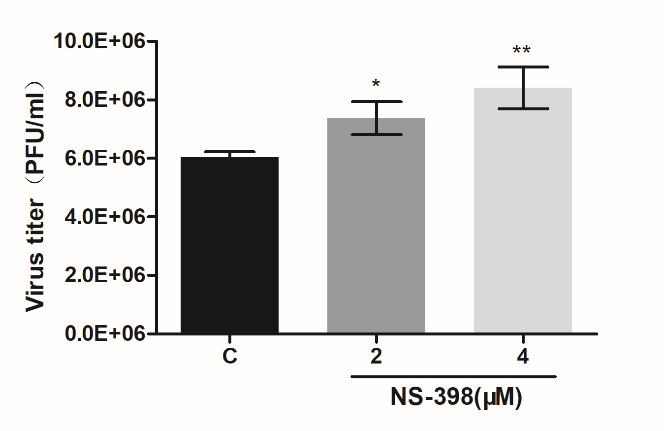


**Figure S1**. **Enhanced proliferation of NDV through inhibition of COX-2 in BHK-21 cell line.**

BHK-21 cells were infected with the F48E9 (MOI = 0.01) and incubated in the absence or presence of NS-398 for 24 h. The supernatants were harvested in order to determine the virus titer by plaque assay.


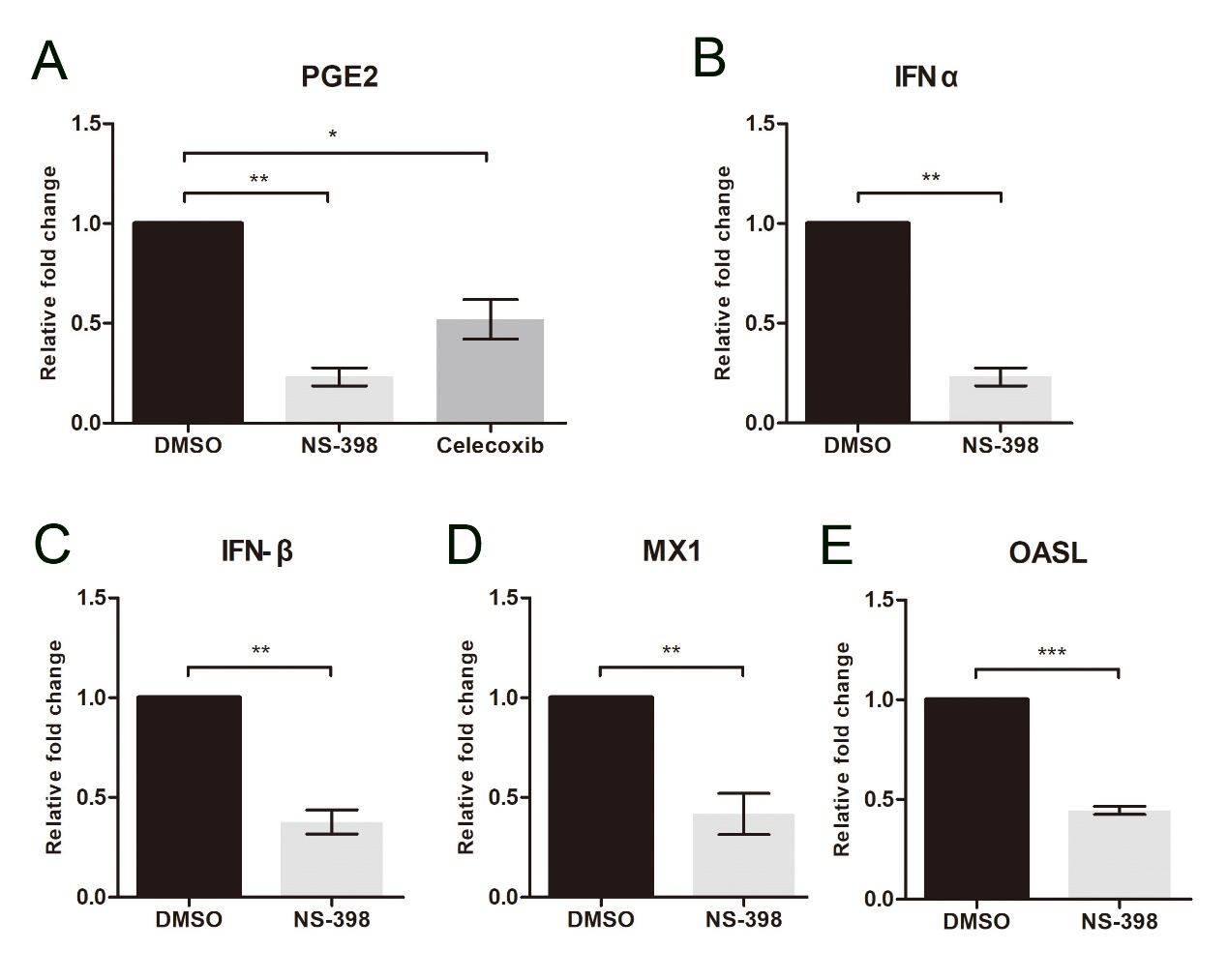


**Figure S2. Effect of NS-398 on DF-1 cells.**

**(A)** DF-1 cells were incubated with NS-398 (4 μM) or celecoxib (2 μM) for 24 h. After the incubation, the supernatants were harvested for the determination of PGE_2_ ELISA. **(B-E)** DF-1 cells were infected with the F48E9 (MOI = 0.01) and incubated with DMSO or NS-398 (4 μM) for 24 h. Cellular mRNA level of (B) IFN-α, (C) IFN-β, (D) MX1 and (E) OASL were analyzed by qPCR.


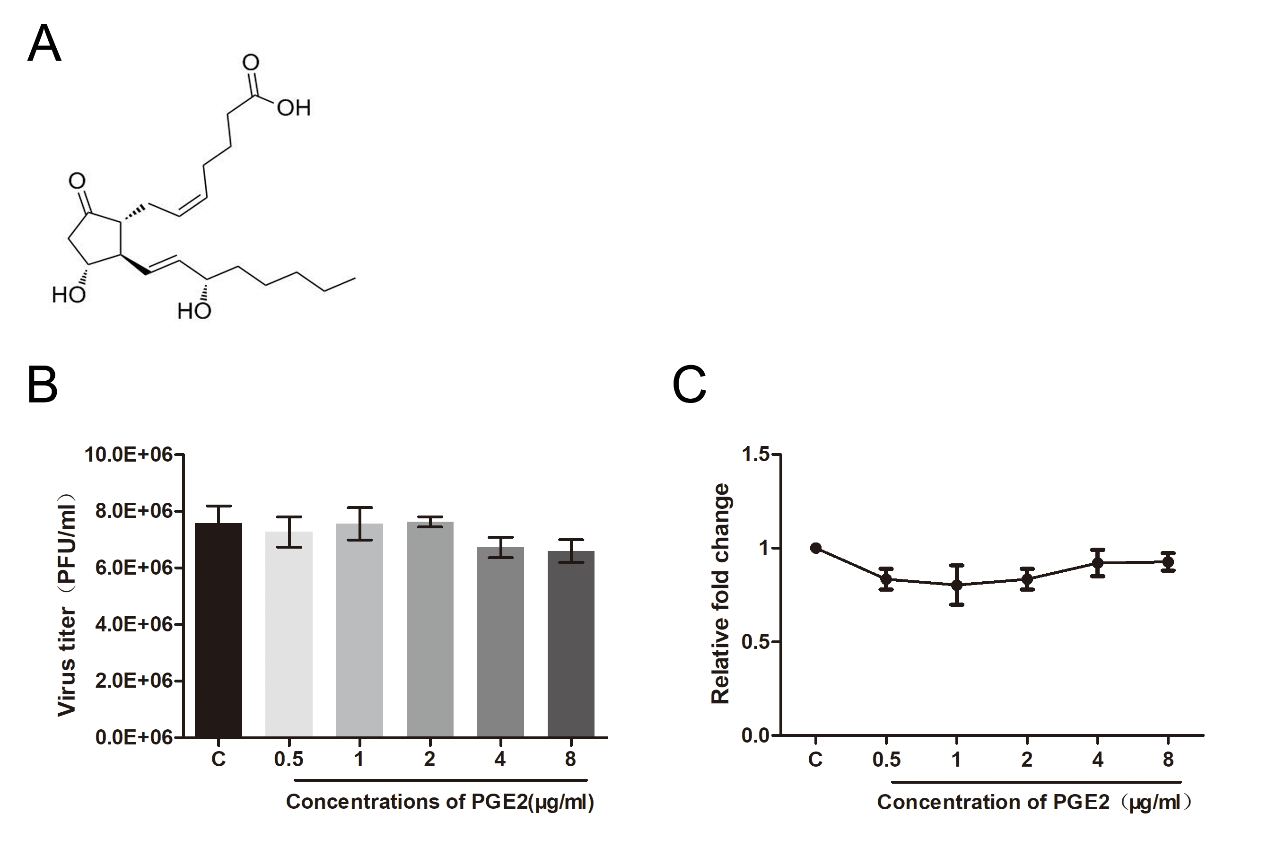


**Figure S3.** **Influence of PGE_2_ on NDV proliferation.**

**(A)** The molecular structure of PGE_2_. **(B, C)** DF-1 cells were infected with the F48E9 (MOI = 0.01) and incubated in the absence or presence of the PGE_2_ for 24 h. The supernatants were harvested in order to determine the virus titer by plaque assay (B). Cellular RNA was extracted, reverse transcribed and analyzed by qPCR (C).


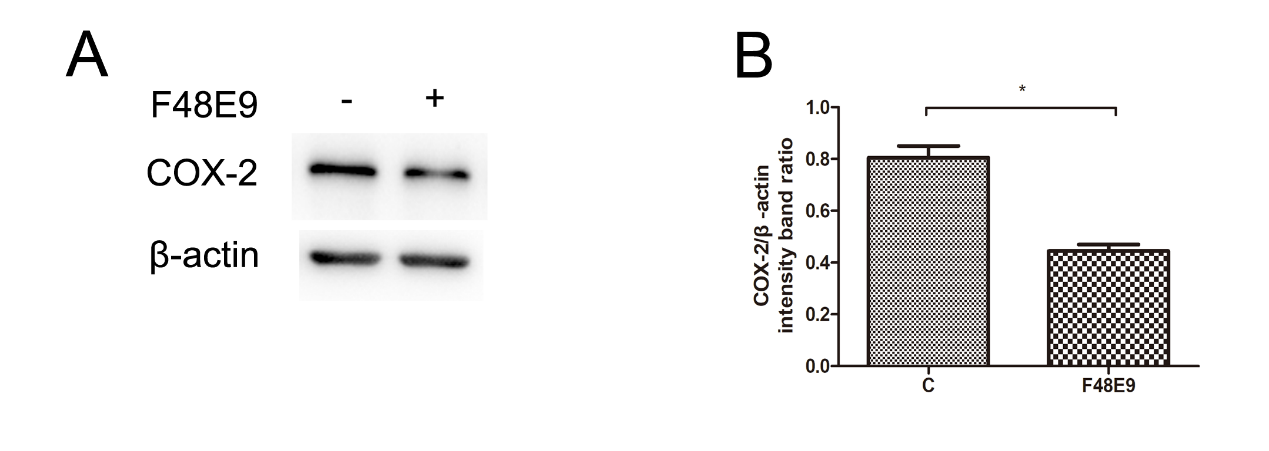


**Figure S4**. **Influence of NDV on COX-2 expression in BHK-21 cell line.**

**(A)** DF-1 were infected with or without F48E9 (MOI=0.01). At 24 h post infection, cell lysates were subjected to western blot. **(B)** Intensity band ratio of COX-2 to β-actin.

The bars indicate the mean ± SD from three independent experiments. SD, standard deviation; ⁎p < 0.05.


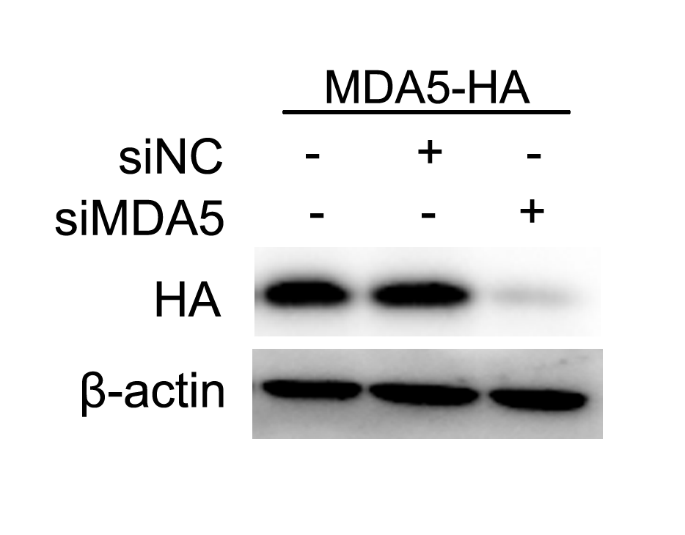


**Figure S5**. **siMDA5 inhibit MDA5 expression.**

DF-1 cells were co-transfected with pCAGGS-MDA5-HA and siMDA5 (or siNC). At 24 hours post transfection, cell lysates were subjected to western blot.
